# Organoiridium Photosensitizers Induce Specific Oxidative Attack on Proteins within Cancer Cells

**DOI:** 10.1002/anie.201709082

**Published:** 2017-10-19

**Authors:** Pingyu Zhang, Cookson K. C. Chiu, Huaiyi Huang, Yuko P. Y. Lam, Abraha Habtemariam, Thomas Malcomson, Martin J. Paterson, Guy J. Clarkson, Peter B. O'Connor, Hui Chao, Peter J. Sadler

**Affiliations:** ^1^ Department of Chemistry University of Warwick Coventry CV4 7AL UK; ^2^ School of Chemistry Sun Yat-Sen University Guangzhou 510275 P. R. China; ^3^ College of Chemistry and Environmental Engineering Shenzhen University Shenzhen 518060 P. R. China; ^4^ Institute of Chemical Sciences Heriot-Watt University Edinburgh EH4 4AS UK

**Keywords:** bioinorganic, cancer, cells, iridium, photosensitizers

## Abstract

Strongly luminescent iridium(III) complexes, [Ir(C,N)_2_(*S*,*S*)]^+^ (**1**) and [Ir(C,N)_2_(O,O)] (**2**), containing C,N (phenylquinoline), O,O (diketonate), or S,S (dithione) chelating ligands, have been characterized by X‐ray crystallography and DFT calculations. Their long phosphorescence lifetimes in living cancer cells give rise to high quantum yields for the generation of ^1^O_2_, with large 2‐photon absorption cross‐sections. **2** is nontoxic to cells, but potently cytotoxic to cancer cells upon brief irradiation with low doses of visible light, and potent at sub‐micromolar doses towards 3D multicellular tumor spheroids with 2‐photon red light. Photoactivation causes oxidative damage to specific histidine residues in the key proteins in aldose reductase and heat‐shock protein‐70 within living cancer cells. The oxidative stress induced by iridium photosensitizers during photoactivation can increase the levels of enzymes involved in the glycolytic pathway.

Selective activation of nontoxic photosensitizers in cancer cells by spatially‐directed light is an attractive regimen for therapy because of the minimal damage to normal cells, especially if the sensitizer is preferentially taken up by cancer cells.^1^ Indeed, this procedure has been widely used in the last 40 years, largely as photodynamic therapy (PDT) in which absorption of light by a photosensitizer (e.g., a porphyrin) promotes formation of a triplet state which can polarize the spins of the ground‐state ^3^O_2_ to generate highly toxic ^1^O_2_. Surface cancers are particularly accessible to PDT, but light can also be directed to a range of other tissues using, for example, fiber optics. Examples of clinical photosensitizers include hematoporphyrin derivatives (Photofrin)^2^ and aminolevulinic acid (ALA, a porphyrin precursor).^3^ Two metal complexes are currently undergoing PDT clinical trials: TLD1433, an octahedral tris‐*N*,*N*‐chelated Ru^II^ complex,^4^ and WST 11, a square‐planar Pd^II^ bacteriochlorophyll derivative.^5^ Such complexes, together with octahedral Ir^III^ complexes, are often luminescent and might be useful in theranostic procedures in which optical imaging is used to monitor the uptake of the sensitizer before subsequent irradiation then kills the cancer cells.^6^

A key advantage for metal complexes is the heavy‐atom effect, which favors fast singlet‐to‐triplet intersystem crossing (ISC), thus giving longer excited‐state lifetimes^7^ and higher yields of ^1^O_2_ and/or other so‐called reactive oxygen species (ROS). The use of red and near IR light (*λ*=600–1100 nm) also enhances the clinical application of PDT because of its low energy compared to either UV or visible light.1d Singlet oxygen is a dominant mediator of photocytotoxic effects, but is short‐lived (<200 ns in vitro).^8^ Consequently, the ^1^O_2_ diffusion distance is short (≈1 μm),^9^ thus limiting cytotoxic damage to the immediate subcellular vicinity of the photosensitizer. Proteins are susceptible to oxidation by photochemically derived ^1^O_2,_ 10 but there are few previous investigations of protein attack by ^1^O_2_ in living cells.6d If photosenstiizers can be distinguished by differences in their oxidative activity towards target sites, then a more rational basis for their use might emerge.

Here we compare the photophysical, chemical, and biological properties of two highly luminescent organoiridium phenylquinoline (C,N) complexes, [Ir(C,N)_2_(*S*,*S*)]^+^ (**1**) and [Ir(C,N)_2_(O,O)] (**2**). Their absorption and emission properties depend on the chelated dithione (*S*,*S*) and diketonate (O,O) ligands, as well as their effectiveness in killing cancer cells in both two‐dimensional (2D) and three‐dimensional (3D) cell cultures, and the ability to generate ^1^O_2_ in cancer cells. Moreover, the photoactivation in cancer cells leads to specific oxidative damage in some key cellular proteins.

The synthesis and characterization of the positively charged Ir^III^ dithione complex **1** and neutral diketonate complex **2** (Figure 1 a) are described in the the Supporting Information. They were isolated as their thermodynamically more‐stable isomers with carbon atoms from the phenylquinolene *trans* to the S atoms of the dithione in **1** and *trans* to the O atoms of the diketonate in **2**. X‐ray crystallography shows that **2** has a distorted octahedral structure (see Figure S1 and Tables S1 and S2 in the Supporting Information) with Ir−O bond lengths of 2.13 to 2.15 Å and an O‐Ir‐O twist angle of 87.18°. Crystals of **1** could not be obtained, but DFT calculations gave Ir‐S bond lengths of 2.58 and 2.56 Å for the most stable CC isomer (see Table S3). In addition, they were highly stable in the cell‐culture medium (RPMI‐1640) for 48 hours (see Figure S2).

**Figure 1:**
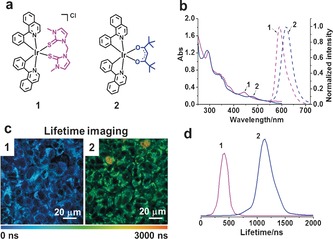
a) Structures of the complexes **1** and **2**. b) Absorption and emission spectra of **1** and **2** in PBS solution (with 2 % DMSO), *λ*_ex_=458 nm. c) PLIM images of living A549 lung cancer cells. d) Lifetimes of **1** and **2** in living A549 cells (*λ*_ex_=458 nm, ƒ=0.5 MHz).

The complex **2** has an MLCT absorption at longer wavelength (*λ*=475 nm) than that of **1** (*λ*=445 nm), and the deep‐red phosphorescence of **2** (*λ*_max_=620 nm) is shifted to longer wavelength compared to that of **1** (*λ*_max_=596 nm; Figure 1 b). TD‐DFT calculations (Figures S3 and S4 and Tables S5 and S6) qualitatively reproduce electronic excitations for both complexes although the primary MLCT absorption band of **2**, compared to **1,** is around 0.1 eV higher, while the phosphorescence emission of **2** is around 0.15 eV higher in energy. The MLCT absorption is blue‐shifted on going from the CC to CN to NN isomers of both **1** and **2**. For all three isomers, the excitation character for the first bright MLCT state is from a d‐character orbital to a π* orbital localized on the phenylquinoline ligand (see Figure S5). All three isomers of **1** support a bound six‐coordinate triplet state (see Table S4). The stability of these triplets follows that of the lowest singlet states: CC as the most stable, CN 6.07 kJ mol^−1^ higher, and NN 37.92 kJ mol^−1^ higher.

The two‐photon luminescence properties of **1** and **2** were investigated by determining the cross‐section *δ* (see Figure S6). The dithione complex **1** exhibited a slightly stronger two‐photon absorption (TPA) at *λ*=750 nm (*δ*_750nm_=115 GM; 1 GM=1×10^−50^ cm^4^ s^−1^ photon^−1^) relative to that of the diketonate complex **2** (*δ*_750nm_=70 GM). The δ values are encouraging for the possible use of **1** and **2** in two‐photon photodynamic therapy, since red light penetrates more deeply into tissues than light with shorter wavelengths.^11^ The two‐photon excitation process was confirmed by its power dependence (see Figure S7). A theoretical investigation of TPA by **1** using a quasi‐parity conserving, three‐state model indicated a very large TPA cross‐section around 740 nm for the CC isomer. This excitation is to a higher lying singlet state (S_7_), which is dark to one‐photon absorption (OPA). The two‐photon absorbing state is of mixed character, with both d‐d, as well as significant MLCT features (see Figure S8). The TPA maximum is shifted to longer wavelengths for the higher energy isomers.

Phosphorescence quantum efficiencies (*Φ*_em_ 0.069–0.097) of the complexes in phosphate‐buffered saline (PBS) were higher under N_2_ than in air (0.032–0.049; see Table S7). Phosphorescence lifetimes (*τ*) of the excited states were much shorter in the presence of O_2_ (241 ns for **1** and 59 ns for **2**) than in the absence of O_2_ (389 ns for **1**, 109 ns for **2**) in PBS (Figure S9). These results suggest that ground‐state ^3^O_2_ interacts with the triplet excited states of these complexes.

The lifetimes of **1** and **2** in living A549 human lung cancer cells were determined to be 404±23 and 1136±72 ns, respectively, by using confocal and phosphorescence lifetime imaging microscopy (PLIM; Figure 1 c,d). These lifetimes, especially that of **2**, are about 19 times longer than for aqueous solutions (in air), thus suggesting that the complexes reside in a more hydrophobic and hypoxic environment in living cells, factors known to enhance the phosphorescence lifetimes of photosensitizers.^12^

^1^O_2_ generation by **1** and **2** under *λ*=465 nm (blue) light irradiation was detected by electron paramagnetic resonance (EPR) spectroscopy using 2,2,6,6‐tetramethylpiperidine (TEMP) as a spin‐trap. The characteristic triplet‐of‐triplets for the 2,2,6,6‐tetramethylpiperidine‐1‐oxyl radical was observed under irradiation. An ^1^O_2_ signal was neither observed in the dark nor in control samples under irradiation (see Figure S10a). The quantum yields for ^1^O_2_ generation [Φ(^1^O_2_)] by **1** and **2** upon irradiation with *λ*=465 nm light were determined as 0.73 and 0.81, respectively (see Table S7). These values are much higher than those for the well‐known [Ru(bpy)_3_]^2+^ [Φ(^1^O_2_)=0.22].1b

To demonstrate that these Ir^III^ complexes can produce cellular ^1^O_2_ after irradiation with *λ*=465 nm light or *λ*=750 nm laser, 2D A549 monolayer lung cancer cells and 3D A549 tumor spheroids were incubated with the Ir^III^ complexes and the fluorescence probe 2,7‐dichlorodihydro‐fluorescein diacetate (DCFH‐DA). Cells treated with only the DCFH‐DA control or the iridium complexes in the dark showed no enhancement of fluorescence. In contrast, a significant increase in fluorescence from DCFH‐DA was observed following light irradiation of the cells treated with either **1** or **2** (see Figures S10 and S11). These findings suggest that **1** and **2** generate ^1^O_2_ efficiently in cancer cells upon light irradiation.

PDT in cell experiments was first tested by incubating monolayer cancer cells with the compounds at various concentrations for 2 hours. There was no loss of cell viability after irradiation in the absence of Ir complexes (control + irradiation; see Figure S12). In the dark, **2** was nontoxic to both A549 lung cancer and MRC‐5 normal lung fibroblasts (IC_50_>100 μm), while **1** showed moderate toxicity to A549 cells (IC_50_=21.2 μm; see Table S8). The potency of both **1** and **2** towards cancer cells increased markedly upon irradiation, notably sub‐micromolar for A549 cells. The phototoxicity index (PI) of **2** towards to A549 cancer cells is greater than 333. Under the same experimental conditions, 5‐ALA and cisplatin displayed much lower phototoxicity (IC_50_>100 μm).

The 2‐photon (2P) photocytotoxicities of **1** and **2** towards 3D multicellular tumor spheroids (MCTSs) were further investigated. Untreated MCTSs were unaffected by 1P and 2P irradiation (see Figure S12). The complex **2** showed no toxicity towards A549 MCTSs in the dark, and also no toxicity towards MRC‐5 MCTSs in either the dark or upon irradiation. However, the IC_50_ values for **2** towards A549 spheroids upon 1P and 2P irradiation were very low, 1.0 μm and 0.23 μm, respectively (see Table S8). Interestingly, 2P photocytotoxicity is higher than 1P photocytotoxicity. Although **1** showed good phototoxicity to A549 spheroids, it was not so selective, thus exhibiting some phototoxicity towards the MRC‐5 spheroids.

The cellular uptake of either **1** or **2** in 3D A549 spheroids was further characterized by 2‐photon confocal laser scanning microscopy. Strong red luminescence was observed on the surface of spheroids to a depth of about 130 μm with *λ*=458 nm excitation and down to about 212 μm with *λ*=750 nm excitation. The luminescence images were captured every 16.3 μm along the *z*‐axis. The spheroids exhibited much stronger luminescence in deeper layers of cells with 2P excitation, thus indicating deeper penetration of the 2P light (Figure 2; and Figures S13 and S14).

**Figure 2:**
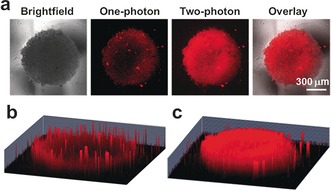
Phosphorescence imaging of A549 spheroids. The spheroids were incubated with **2** (10 μm) for 2 h. a) Comparison of brightfield, one‐photon (*λ*_ex_=458 nm), and two‐photon (*λ*_ex_=750 nm) excitation, *λ*_em_=620±30 nm. 1P (b) and 2P (c) 3D Z‐stack images were taken every 16.3 μm from the top to bottom of the spheroids. Images were taken under a 10× objective. Scale bar: 300 μm.

A549 cancer cells (ca. 1×10^9^) were treated with **2** (10 μm, 2 h) either in the dark or with *λ*=465 nm light irradiation. Proteins from these cells were collected and digested as described in the Supporting Information. LC‐MS/MS data were searched for oxidative modifications of the side chains of cysteine, histidine, tyrosine, phenylalanine, methionine, and tryptophan, as well as asparagine and glutamine deamidation and serine, threonine, and tyrosine phosphorylation (see the Supporting Information). With 1 % false discovery rate (FDR) against the database, only proteins which presented three times or more (in triplicates) under all three conditions (control, drug‐treated with no irradiation, drug‐treated with irradiation) were quantified. 212 proteins and 1500 peptides met these requirements and were identified. Two‐tail t‐tests ensured the consistency of the reference lysozyme peptide (FESNFNTQATNR, 714.8365 *m*/*z*) throughout all samples (see Table S9). All p values were calculated to be larger than 0.05 (i.e., quantities of the reference lysozyme peptide were statistically similar, and consistent throughout all data sets). Hence, the oxidized peptide/lysozyme peptide area ratios were calculated using t‐tests to analyze the significance of changes in quantities of the oxidized peptides under different conditions. The levels of five oxidized peptides (detected three times or more out of the five replicates) were significantly different (see Table S10). These peptides were quantified against the standard lysozyme peptide as described in the Supporting Information. Two unique oxidized peptides have p values of less than 0.05 (significantly different; see Table S10 and Figure 3) and were upregulated. The level of oxidized peptide Ala329‐Arg442, AQIHDIVLVGGSTR (*m*/*z* 741.4156; Figure 3 a; see Table S11), from the 70 kDa heat‐shock protein (Hsp 70), increases by 5.8‐fold for drug‐treated cells upon irradiation compared to the drug‐treated cells in the dark. Specific oxidation of His332 to 2‐oxo‐His332 was identified by LC‐FT‐ICR MS/MS. The second oxidized peptide, Tyr178‐Lys195, from aldose reductase, YKPAVNQIECHPYLTQEK (*m*/*z* 745.3780; with alkylated Cys, Figure 3 c; see Table S12) contained 2‐oxo‐His188, which increased by 3.0‐fold for drug‐treated cells upon irradiation as compared to that in the dark (Figure 3; see Table S10). These data appear to be the first report of the formation of 2‐oxo‐His^13^ after treatment of cancer cells with organometallic photosensitizers.

**Figure 3:**
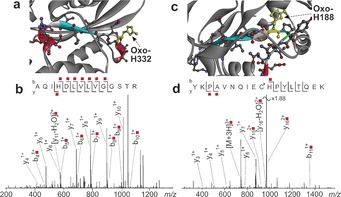
a) Structure of Hsp 70 (PDB:3ATV),^14^ with the oxidized peptide Ala329‐Arg342 shown in color (2‐oxo‐His332, yellow). b) LC MS/MS (CAD) of the oxidized peptide from HSP70. c) Structure of aldose reductase (PDB:1US0),^15^ with the oxidized peptide Tyr178‐Lys195 shown in color (2‐oxo‐His188, yellow). d) LC MS/MS (CAD) of the oxidized peptide from aldose reductase. Fragments with red labels indicate the presence of oxidation, and an asterisk indicates alkylated Cys.

Pathway analysis was carried out to investigate the overall effects induced by **2** on cell metabolism (for methodology,^16^ see the Supporting Information). The most significant result identified nine unique proteins along the glycolysis pathway (see Table S13 and Figure S15). The levels of these proteins, which are all involved in the conversion of glucose to pyruvate, increased by factors of about 2.1–5.3‐fold on irradiation of A549 cancer cells treated with **2**, with the highest increase for fructose‐bisphosphate aldolase. Cancer cells have defective mitochondria and increase their rate of glycolysis as a source ATP and energy to compensate for this mitochondrial effect. Mitochondria, where oxygen is reduced to water, are also a major source of ROS in cells.^17^ During irradiation, a vast amount of ^1^O_2_ is generated and, a loop of ROS‐stimulated glucose uptake and glucose‐stimulated ROS production is triggered.^18^ This process is consistent with the up‐regulation of proteins in the glycolytic pathway.

In summary, we designed efficient new organoiridium photocatalytic sensitizers which were nontoxic in the dark and highly and selectively cytotoxic to cancer cells when irradiated by 1P and 2P irradiation (especially complex **2**) in the screening against 2D and 3D (spheroid) cancer cell models. In previous reports, the specific nature of the damage to proteins in the cell, induced by photodynamic therapy, has been little studied. We found that ^1^O_2_ generated by **2** can oxidize specific histidines in the proteins Hsp 70 and aldose reductase (AR), which have important functions in cancer cells. Hsp 70 is a molecular chaperone for nascent proteins, and aberrantly folded, damaged, or mutated proteins and AR is a monomeric reduced nicotinamide adenine dinucleotide phosphate (NADPH)‐dependent enzyme, a member of the aldo‐keto reductase superfamily. This work appears to be the first report showing that specific sites of cellular Hsp‐70 and AR can be oxidized during PDT. The combination of oxidative stress induced by the photoactivation of **2** together with the malfunction of mitochondria in cancer cells leads to the increased use of glucose to generate energy, and is consistent with the observed increase in the levels of all enzymes involved in the glycolytic pathway (by factors of about 2.1 to 5.3‐fold).

## Conflict of interest

The authors declare no conflict of interest.

## Supporting information

As a service to our authors and readers, this journal provides supporting information supplied by the authors. Such materials are peer reviewed and may be re‐organized for online delivery, but are not copy‐edited or typeset. Technical support issues arising from supporting information (other than missing files) should be addressed to the authors.

Supplementary
