# Supplementary material for: Organoiridium Photosensitizers Induce Specific Oxidative Attack on Proteins within Cancer Cells
Source: Angew Chem Int Ed Engl. 2017 Oct 19;56(47):14898–902. doi: 10.1002/anie.201709082 (PMC5698709; doi:10.1002/anie.201709082)
Supplement: Supplementary file 1 — Supplementary [file ANIE-56-14898-s001.pdf]

Angewandte Chemie
Eine Zeitschrift der Gesellschaft Deutscher Chemiker

Supporting Information

Organoiridium Photosensitizers Induce Specific Oxidative Attack on Proteins within Cancer Cells

Pingyu Zhang[+], Cookson K. C. Chiu[+], Huaiyi Huang, Yuko P. Y. Lam, Abraha Habtemariam, Thomas Malcomson, Martin J. Paterson, Guy J. Clarkson, Peter B. O'Connor, Hui Chao,* and Peter J. Sadler**

anie_201709082_sm_miscellaneous_information.pdf

Experimental
ES1 Materials
ES2 Synthesis
ES3 Experimental methods
Table S1. Crystal data and structure refinement for **2**.
Table S2. Selected bond lengths (Å) and angles (°) for **2**.
Tables S3-4. Selected geometrical parameters for **1** isomers in the singlet and triplet states.
Table S5. TD-DFT one-photon excitation data for **1** (CC isomer).
Table S6. TD-DFT one-photon excitation data for **2** (CC isomer).
Table S7. Photophysical data for the complexes.
Table S8. (Photo)cytotoxicity of the compounds towards 2D and 3D cells.
Table S9. T-test results for lysozyme peptide.
Table S10. Identification and changes in cellular levels of oxidized peptides
Table S11. Mass error table for Heat Shock Protein 70 kDa peptide (Ala329-Arg342).
Table S12. Mass error table for Aldose Reductase peptide (Tyr178-Lys195).
Table S13. Quantification of glycolysis pathway.
Fig. S1. X-ray crystal structure of complex **2**, 400 MHz ^{1}H NMR and mass spectra of complexes **1** and **2**.
Fig. S2. The stability of **1** and **2** in cell culture medium for 48 h.
Fig. S3. Simulated electronic spectrum for **1** (CC isomer).
Fig. S4. Simulated electronic spectrum for **2** (CC isomer).
Fig. S5. Dominant canonical particle-hole orbitals characterising the two lowest singlets and first triplet state of **1** (CC isomer).
Fig. S6. Two-photon absorption of the Ir complexes between 720 and 800 nm.
Fig. S7. Logarithmic two-photon luminescence intensity vs the power of irradiation.
Fig. S8. Dominant canonical particle-hole orbital representation of the TPA state (S_7) of **1** (CC isomer).
Fig. S9. Phosphorescence lifetimes of the Ir complexes.
Fig. S10. Singlet oxygen detection by EPR and fluorescence imaging.
Fig. S11. ROS measurements in cells.
Fig. S12. Cell viability of the control cells after irradiation.
Fig. S13. Z-stack images of 3D tumor spheroids matched in Fig. 2.
Fig. S14. One-photon and two-photon images of 3D tumor spheroids treated with complex **1**.
Fig. S15. Quantification of glycolysis pathway.
References

Experimental

Materials. 2,2,6,6-Tetramethyl-3,5-heptanedione, 2-ethoxyethanol, rhodamine B, 2′,7′-dichlorofluorescin diacetate (H_2DCF-DA), DMSO-d_6 and cisplatin were purchased from Sigma-Aldrich. Human lung carcinoma (A549) cell line was purchased from ECACC. MRC-5 (human normal lung fibroblast) cell line was purchased from Sigma-Aldrich. Roswell Park Memorial Institute medium (RPMI-1640), fetal calf serum (FCS), glutamine and penicillin/streptomycin were purchased from Sigma-Aldrich. The dinuclear iridium complex [(1pq)$_4$Ir$_2$Cl$_2$)]) was prepared according to literature methods [1].

Instruments. ^{1}H NMR spectra were recorded on a BrukerAV-400 spectrometer. Elemental analysis was performed by Exeter Analytical using a CHN/O/S Elemental Analyser (CE440). Positive ion ESI-MS spectra were obtained using an Agilent 6130B single quad coupled to an automated sample delivery system (isocratic Agilent 1100 HPLC without column). UV-visible absorption spectra were recorded on a Varian Cary 300UV-vis spectrophotometer fitted with an external Varian Cary temperature controller. The fluorescence spectra were recorded on a JASCO FP-6500 Fluorimeter. The Ir(III) complexes were tested against cancer cells using the 96-array of LEDs (blue, λ_{irr} = 465 nm, 4.8 mW cm^{-2}). The 96-array LEDs were constructed by Mr Rodney Wesson, Electrical and Electronics Workshop, Department of Chemistry, University of Warwick.

Synthesis

Synthesis of the dithione ligand. 1-Methyl-1H-imidazole-2-thiol (60 mmol) in CH_2Cl_2 (20 mL) was heated for 24 h at 353 K in a sealed tube. The solution was cooled to ambient temperature, and the solvent was removed under reduced pressure. The solid was redissoved in CH_3OH (5 mL) and 100 mL tetra-hydrofuran (THF) was added, and the mixture was stirred for 2 h. The precipitate formed was collected by filteration and washed with THF. Yield: 89%. ESI-MS: 241[Mr+H]$^+$. ^{1}H NMR (400 MHz, $CDCl_3$): δ = 7.53 (d, J = 7.6 Hz, 2H), 7.46 (d, J = 8.5 Hz, 2H), 5.02 (s, 2H), 3.27 (s, 6H). Anal. Calcd for $C_9H_{12}N_4S_2$: C, 44.98%; H, 5.03%; N, 23.31%; S, 26.68 %. Found: C, 45.05%; H, 5.01%; N, 23.28%; S, 26.64%.

Synthesis of complexes 1 and 2. A mixture containing the dithione (0.15 mmol), Ag_2O (0.15 mmol), the iridium dimer (0.07 mmol) in CH_2Cl_2 (50 mL) was heated overnight at 348 K in a sealed tube. After cooling to ambient temperature, the mixture was filtered through celite and washed with CH_2Cl_2. The filtrate was concentrated under reduced pressure and purified on a silica column by Biotage flash chromatography with CH_2Cl_2:CH_3OH (v/v 80:20) as eluant.

Complex **2** was synthesized in a similar manner, but the exclusion of Ag_2O and shorter reaction time (2 h). Briefly, a mixture containing the diketonate ligand (0.15 mmol), the iridium dimer (0.07 mmol) and 50 mL CH_2Cl_2 was heated for 2 h in a seal tube. Then the mixture was

concentrated under reduced pressure and purified on a silica column by Biotage flash chromatography using CH_2Cl_2:CH_3OH (v/v 95:5) as eluant.

Complex 1: Yield: 78%. ESI-MS: 841.3 (M[**1**-Cl]$^+$). ^{1}H NMR (400 MHz, DMSO-d^6): δ 8.86 (dd, *J* = 7.5, 4.9 Hz, 4 H), 8.14 (dd, *J* = 11.1, 8.5 Hz, 4 H), 7.94 – 7.86 (m, 2 H), 7.83 (t, *J* = 8.5 Hz, 4 H), 7.56 (d, *J* = 1.3 Hz, 2 H), 7.01–6.87 (m, 4 H), 6.81 (t, *J* = 7.4 Hz, 2 H), 6.19 (d, *J* = 7.4 Hz, 2 H), 4.25 (s, 2 H), 3.72 (s, 6 H). Anal. Calcd for $C_{39}H_{32}IrN_6S_2Cl$: C, 53.44%; H, 3.68%; N, 9.59%; S, 7.32%. Found: C, 53.49%; H, 3.67%; N, 9.53%; S, 7.30%.

Complex 2: Yield: 92%. ESI-MS: 785.4 (M[**2**]), 808.4 M ([**2**+Na]$^+$). ^{1}H NMR (400 MHz, $CDCl_3$): δ 8.93 (dd, *J* = 6.2, 3.6 Hz, 2H), 8.27 (d, *J* = 6.4 Hz, 2H), 8.14 (d, *J* = 7.9 Hz, 2H), 7.83 (dd, *J* = 6.1, 3.4 Hz, 2H), 7.67 – 7.57 (m, 4H), 7.33 (s, 2H), 6.83 (t, *J* = 7.5 Hz, 2H), 6.59 (t, *J* = 7.3 Hz, 2H), 6.44 (d, *J* = 7.5 Hz, 2H), 5.35 (s, 1H), 0.74 (s, 18H). Anal. Calcd for $C_{41}H_{39}IrN_2O_2$: C, 62.81%; H, 5.01%; N, 3.57%. Found: C, 62.88%; H, 4.97%; N, 3.59%. Crystals suitable for X-ray diffraction were obtained by evaporation of a CH_2Cl_2 solution at ambient temperature.

X-ray Crystal Structure. A suitable crystal was selected and mounted on a glass fibre with Fromblin oil and placed on an Xcalibur Gemini diffractometer with a Ruby CCD area detector. The crystal was kept at 150(2) K during data collection. Using Olex2[2], the structure was solved with the ShelXT[3] structure solution program using Direct Methods and refined with the ShelXL[3] refinement package using Least Squares minimisation. X-ray crystallographic data for **2** have been deposited in the Cambridge Crystallographic Data Centre under the accession number CCDC 1550488.

DFT Calculations. The lowest singlet and triplet state geometry optimisations were performed using a range of functionals (BP86, B3LYP, M06, CAM-B3LYP, PBE0). For the singlet states only very minor differences with each functional are observed, with slightly more variance seen in triplet geometries. The results in Tables S3 and S4 are for BP86 and quoted in the main text as these have been shown to generally give reliable singlet-triplet energetics in a range of molecules. Tables S3 and S4 show selected geometrical parameters for each isomer in the singlet and triplet states. Several one-electron basis sets were also compared and calibrated, with results quoted for the 6-311G(d,p) basis on the non-metal atoms, coupled with a Stuttgart-Dresden (SDD) effective core potential for the iridium (60 core electrons) with a standard SDD polarized double zeta valence set on the metal. Minima were confirmed by noting a positive definite geometrical Hessian matrix obtained by analytical differentiation. For time-dependent (TD) linear response calculations several functionals were again compared with the B3LYP

functional chosen as it gave the best agreement with experimental band maxima. For the TD-DFT calculations the larger def2-TZVPP basis set was used for the metal valence electrons. The TD calculations were run for the first 30 singlet and triplet states. Tables S5 and S6 show the excitation energies, oscillator strengths, and characterization for the lowest excited states for the CC isomers of **1** and **2**. Figs. S3 and S4 show a simulated one-photon absorption spectrum for the CC isomers of **1** and **2**, while Fig. S5 shows canonical particle-hole orbital characterization of the lowest singlet and triplet states of **1**, as obtained from the response eigenvectors. Two-photon absorption was calculated using the 3- and 4-state sum-over-states models of Ågren et al.,[4] using the linear response ground to excited transition moments, and the approximate excited to excited transition moments obtained from the *a posteriori* Tamm–Dancoff approximation (ATDA)[5, 6]. Fig. S8 shows the dominant canonical particle-hole orbital representation of the strong TPA state (S_7) of **1**. All calculations were performed using the Gaussian09 program suite[7].

Determination of 2-Photon Absorption Cross Sections. The 2-photon absorption spectra of probes were determined over a broad spectral range by a typical 2-photon induced luminescence (TPL) method using Rhodamine B in methanol as a standard. The 2-photon luminescence data were acquired using an Opolette™ 355II (pulse width ≤ 100 fs, 80 MHz repetition rate, tuning range 720-800 nm, Spectra Physics, Inc., USA). 2-photon luminescence measurements were performed in fluorometric quartz cuvettes. The experimental luminescence excitation and detection conditions were conducted with negligible reabsorption processes, which can affect the TPA measurements. The quadratic dependence of 2-photon-induced luminescence intensity on the excitation power was verified at an excitation wavelength of 808 nm. The 2-photon absorption cross section of the probes was calculated at each wavelength according to Eq. (1):

$$\delta_2 = \delta_1 \frac{\phi_1 C_1 I_2 n_2}{\phi_2 C_2 I_1 n_1} \quad (1)$$

where I is the integrated luminescence intensity, C is the concentration, n is the refractive index, and Φ is the quantum yield. Subscript '1' indicates reference samples, and '2' indicates experimental samples.

Quantum Yields of Singlet Oxygen. The quantum yields of singlet oxygen were determined using two different methods[8].

Indirect Method. An air-saturated PBS buffer solution, containing the complex (OD = 0.1 at irradiation wavelength), p-nitrosodimethyl aniline (RNO, 20 μM), histidine (10 mM) were irradiated with blue light in a quartz cuvette for different time intervals. The absorbance of the solution was then recorded. Plots of variations in absorbance at 440 nm in PBS (A_0–A, where A_0 is the absorbance before irradiation) versus the irradiation times for each sample were

prepared and the slope of the linear regression was calculated (S_{sample}). As a reference compound, $[Ru(bpy)_3]^{2+}$ (Φ_{ref} ($^1O_2$) = 0.22 in H_2O) was used in both methods, to obtain S_{ref}. Equation (2) was applied to calculate the singlet oxygen quantum yields (Φ_{sample}) for every sample: I (the absorbance correction factor) was obtained using Equation (3), where I_0 is the light intensity of the irradiation source in the irradiation interval and A_λ is the absorbance of the sample at wavelength λ.

$\Phi_{sample} = \Phi_{ref} * S_{sample} / S_{ref} * I_{ref} / I_{sample}$ (2)

$I = I_0 * (1 - 10^{-A\lambda})$ (3)

Direct Method (near-infrared luminescence). Singlet oxygen production by the complexes was measured when the absorbance reached approximately 0.2 at the irradiation wavelength. This solution was then irradiated in quartz fluorescence cuvettes (width 1 cm) using a combined fluorescence lifetime and steady state spectrometer (Edinburgh Instruments Ltd) with lamps of different wavelengths. Singlet oxygen near-IR luminescence at 1270 nm was measured by recording spectra from 1050 to 1500 nm. The intensity of irradiation was varied via neutral density filters. Singlet oxygen luminescence peaks at different irradiation intensities were integrated and the resulting areas were plotted vs. irradiation intensities. The quantum yields were then calculated by applying the same formulas as the indirect method.

Electron Paramagnetic Resonance (EPR). The EPR measurements were carried out at ambient temperature on a Bruker EMX spectrometer. Irradiation was carried out with a 465 nm blue LED. The samples were contained in a flat-cell (WG812) positioned in a TM110 cavity (ER4103 TM). For kinetic measurements, the EPR parameters were: sweep width 8 mT, 1024 points, time constant 10.24 ms and conversion time 20.48 ms, giving a sweep time of ~30 s. Field modulation was applied at 100 kHz and 0.05 mT, and the microwave attenuation was 18 dB (~3.2 mW). The spin trap, 2,2,6,6-tetramethyl-piperidine (TEMP for trapping $^1O_2$, 20 mM), was used to verify the formation of $^1O_2$ generated by the iridium complexes (100 μM).

Monolayer Cell Culture. The cells were grown in RPMI-1640 with or without phenol red (for photoactivable experiment). All media were supplemented with 10% v/v of fetal calf serum (FCS), 1% v/v of 2 mM glutamine and 1% v/v penicillin/streptomycin. All cells were grown as adherent monolayers at 310 K in a 5% CO_2 humidified incubator and passaged regularly at approx. 80% confluence.

Phosphorescence Lifetime Imaging (PLIM). A549 cells were treated with the iridium complexes (5 μM) for 1 h at 37 °C followed by 2× washing with PBS. Then 1 mL of PBS was added and the phosphorescence lifetime images of living cells were recorded by PLIM (setup integrated with an Olympus FV1000 laser scanning confocal microscope; λ_{ex} = 458 nm, λ_{em} = 620 ± 30 nm). Lifetimes were calculated using PicoQuant Co. software.

Cellular $^1O_2$ Measurement. The intracellular ROS under irradiation was measured using the fluorescent probe 2′,7′-dichlorofluorescein diacetate (DCFH-DA). DCFH-DA diffuses through the cell membrane and is enzymatically hydrolyzed by intracellular esterases to form the non-fluorescent compound DCFH, which is then rapidly oxidized to form the highly fluorescent 2′,7′-dichlorofluorescein (DCF) in the presence of ROS. The DCF fluorescence intensity is thought to parallel the amount of ROS formed intracellularly. Firstly, the cultured cancer cells were treated with the iridium complexes in the dark for 2 h, the 2D cells were washed with PBS and then incubated with 10 μM of DCFH-DA for 30 min (1 h for spheroids) in the dark. The cells were subjected to 465 nm light and 750 nm 2-photon laser irradiation for 10 min and then imaged by confocal microscopy immediately. The excitation wavelength of intracellular ROS was set as 488 nm, and the fluorescence was collected at 530 ± 20 nm. The intensity of the fluorescence was measured on a microplate reader (Promega).

Photocytotoxicity for Monolayer Cells. The photocytotoxicity was determined in A549 lung cancer cells and normal MRC-5 lung cells. Approximately 5×10^3 cells/well were seeded into two 96-well plates (‘dark’ and ‘light’ plates), followed by 24 h incubation for attachment. All cells were then exposed to the iridium complexes with different concentrations. After 2 h incubation, each well of both plates was washed with phosphate-buffered saline (PBS), and fresh medium was added into the wells. Cells of irradiated plate were then irradiated (465 nm, 4.8 mW J/cm^2, 10 min). After irradiation, cell incubation was continued for another 46 h. The ‘dark’ plate was treated similarly but without irradiation. The photocytotoxicity was measured by the standard MTT method. The change in optical density (OD) at 540 nm was monitored using a microplate reader (Promega).

2-Photon Photocytotoxicity for Multicellular Tumor Spheroids. MCTSs were cultured using the liquid overlay method. A549 and MRC cells in the exponential growth phase were dissociated by a trypsin/EDTA to provide single-cell suspensions. 10,000 diluted cells were transferred to U-shaped 96-well plates in 200 μL of medium. The single cells generated spheroids ca. 400 μm in semidiameter at day 3 in 5% CO_2 at 37°C. After formation, each spheroid in the 96-well plate was imaged with a phase contrast microscope (10× objective, Zeiss, Germany) to monitor its integrity and semidiameter.

The spheroid-containing medium was replaced carefully with drug-supplemented medium (iridium complexes, ALA or cisplatin) using an eight-channel pipette. Four spheroids were treated per condition and drug concentration, and the DMSO volume was less than 1% (v/v). After incubation in the dark for 2 h, the medium was replaced by fresh medium. The MCTSs in ‘light’ plate were exposed to irradiation (465 nm, 2.88 J/cm^2 or 750 nm, 10 J/cm^2, 100 fs), then incubation of the MCTSs continued for another 46 h. The ‘dark’ plate was under the same

treatment except irradiation. The cytotoxicity of iridium complexes toward MCTSs was measured by ATP concentration with CellTiter-Glo® 3D Cell Viability Assay (Promega). Briefly, an equivalent volume of CellTiter-Glo® 3D reagent was added to each MCTS sample in 96-wells plate; after 5 min shaking and 25 min incubation, the intensity of chemiluminescence was recorded on the Promega microplate reader.

2-Photon Imaging of MCTSs. The spheroids were treated with the iridium complexes (10 μM) for 2 h. Then they were imaged using a Zeiss confocal laser scanning microscope (10× objective). For 1-photon images, the excitation wavelength of the laser was 458 nm. For 2-photon images, the excitation wavelength of the laser was 750 nm. The wavelength of emission was 620 ± 30 nm.

Proteomics for Target Site Identification. Around 1×10^9 A549 cancer cells were treated with complex **2** (10 μM) in the dark or with 10 min of LED irradiation (465 nm). All cells were harvested and lysed by sonicating with glass beads at 277 K in a Tris-buffer solution (50 mM Tris-HCl with 1% glycerol; pH 7.5). The samples were sonicated for 30 s and cooled for 5 min, the process being repeated 10 times. All samples were then centrifuged for 30 min at 5,000 g at 277 K to remove the pellets. The supernatant from each sample was then quantified against a standard calibration curve of bovine serum albumin (BSA) using Bradford Reagent (Sigma Aldrich Company Ltd; Dorset, England). The concentrations of proteins in these samples were then calculated.

The above samples were diluted using 100 mM ammonium bicarbonate solution (Sigma Aldrich Company Ltd; Dorset, England); the disulfide bonds were reduced with 50 mM dithiothreitol (Sigma Aldrich Company Ltd; Dorset, England) and the free Cys thiols alkylated with 100 mM iodoacetamide (Sigma Aldrich Company Ltd; Dorset, England). The alkylated lysate was then digested with trypsin (Sigma Aldrich Company Ltd; Dorset, England) by incubating the mixture for 16 h at 310 K. The digested peptides were desalted using C18 SPE cartridges (Thermo Scientific), washed twice with Milli-Q water and then eluted with 80% ACN/aqueous solution. Desalted samples were dried down with Speed Vac concentrator (Thermo Scientific) and re-dissolved in Milli-Q water into concentration of 1 mg/mL.

Nano-LC separations were achieved using an EASY nano-LC II system (Proxeon, Hemel Hempstead, UK) with a home-made 18 cm, C18 reverse phase (RP) nano capillary column (75 µm, 5 µm particle size) and a 3 cm C18 RP pre-column (150 µm, 5 µm particle size). Separation of tryptic cell digest was achieved using an acidified water/acetonitrile gradient from 5% ACN to 30% ACN over 120 minutes, followed by a second gradient of 15 minutes from 30% ACN to 80% ACN. Finally, with a 35-min wash of 80% ACN at a constant 600 nL/min flow rate.

LC-MS/MS experiments were carried out by coupling EASY-nLC to SolariX FT-ICR Mass Spectrometer, via a custom nano-spray source utilising glass nanospray tips, 360 µm tip O.D.,

50 μm tip I.D., and 8 μm orifice I.D. (New Objective, MA, USA). Singly charged or unknown charged species were excluded automatically from the MS/MS list, only the most intense multiply charged ions from each scan will be isolated in the quadrupole and accumulated for 1.2 s in the hexapole collision cell for collisionally activated dissociation (CAD) MS/MS experiments. 1.6 μg of each sample was injected for each LC-MS/MS run. All LC-MS/MS experiments were done with 5 replicates for consistency and reproducibility, and more importantly, to increase the reliability of the data.

Mass lists were generated by the FTMS peak picking algorithm in Data Analysis v4.2 (Bruker Daltonik GmbH, Bremen, Germany) from LC-MS/MS data sets. All the identified proteins were filtered with 1% false discovery rate (FDR). Each oxidized peptide picked out by the search engine was manually analysed from the raw LC-MS/MS data, and any false positive results were critically eliminated. The reduced datasets (after elimination), were then grouped together, and oxidized peptides that were detected 3 times or more out of the 5 replicates of data were further selected for quantification.

Singlet-oxygen-induced oxidation of proteins is complicated as discussed in several reviews[9,10], being the result of direct attack on amino acids and secondary attack by initially formed peroxides. On the basis of previous reports, the following modifications were included in the search:

Variable modifications: Met – sulfoxide, sulfone; His – 2-oxo-histidine; Trp – kynurenine, 3-hydroxykynurenine, N-formylkynurenine; Cys – sulfenic acid, sulfinic acid, sulfonic acid; Phe, Tyr – dihydroxyphenylalanine.

In addition, the fixed carbamidomethyl modification of Cys (from alkylation) and variable deamidation modifications of Asn, Gln and phosphorylation of Ser, Thr and Tyr were included.

Quantification of peptides was made by spiking with a lysozyme peptide in 1:6 lysozyme peptide:cell digest volume ratio (lysozyme peptide 0.01 μg/μL, cell digest 0.2 μg/μL) into each sample during the LC-MS/MS runs, and by determining the ratio between the cell peptide of interest and the specific lysozyme peptide. The differences between the ratios from the control sample set and the drug treated sample set were compared. T tests and p values were determined as measures of the level of significance between the different samples.

Pathway Analysis. Peptides that were detected 3 times or more out of the 5 replicates of data were selected for quantification of pathway analysis. The protein entry lists were imported and cross-matched with DAVID Bioinformation Database, then a list of related pathways was generated and exported by KEGG database[11-14]. Glycolysis, which was found to have the most number of protein counts and lowest Fisher Exact p-values were chosen for quantification using the methodology described above. The weighted area ratio was calculated using the equation shown below for proteins with multiple identified peptides. This provided a more comprehensive measure of the change in abundance under different conditions.

$$Average\ Fold\ of\ Change = \sum F \times \frac{a}{\sum a}$$

F = Fold of change of a peptide; a = average area ratio among the 5 replicates of data sets

Tables

Table S1. Crystal data and structure refinement for **2.**

Complex	**2**
Empirical formula	$C_{41}H_{39}IrN_2O_2$
Formula weight	783.94
Temperature/K	150(2)
Crystal system	monoclinic
Space group	$P2_1/c$
a/Å	10.7708(2)
b/Å	16.1680(3)
c/Å	19.0863(4)
α/°	90
β/°	90.4187(17)
γ/°	90
Volume/Å^3	3323.62(10)
Z	4
ρ_{calc}g/cm^3	1.567
μ/mm^{-1}	4.056
F(000)	1568.0
Crystal size/mm^3	0.34 × 0.1 × 0.1 red block
Radiation	MoKα (λ = 0.71073)
2Θ range for data collection/°	5.032 to 65.692
Index ranges	$-15 \leq h \leq 15$, $-24 \leq k \leq 24$, $-22 \leq l \leq 27$
Reflections collected	41285
Independent reflections	11320 [R_{int} = 0.0371, R_{sigma} = 0.0397]
Data/restraints/parameters	11320/0/421
Goodness-of-fit on F^2	1.055
Final *R* indexes [*I>=2σ (I)]*	R_1 = 0.0305, wR_2 = 0.0633
Final *R* indexes [all data]	R_1 = 0.0464, wR_2 = 0.0705
Largest diff. peak/hole / e Å^{-3}	1.88/-2.06

Table S2. Selected bond lengths (Å) and angles (°) for **2**.

**2**	Bond length/ angle
Ir1-O37	2.134(2)
Ir1- O39	2.151(18)
Ir1- N1	2.037(2)
Ir1-N17	2.028(2)
Ir1- C16	1.988(2)
Ir1-C32	1.975(3)
O37-Ir1-O39	87.18(7)
N1-Ir1-O37	83.31(8)
N1-Ir1-O39	94.05(8)
N17-I r1-O37	96.27(8)
N17-Ir1-O39	90.89(8)
N17-Ir1-N1	175.00(8)
C16-Ir1-O37	91.16(9)
C16-Ir1-O39	173.84(9)
C16- Ir1-N1	79.86(10)
C16-Ir1-N17	95.18(10)
C32-Ir1-C16	92.88(10)
C32-Ir1-O37	174.87(9)
C32-Ir1-O39	89.13(9)
C32-Ir1-N1	100.51(10)
C32-Ir1-N17	80.22(10)

Table S3. Selected bond lengths (Å) and angles (°) for isomers of **1** in the ground singlet state, as optimized with R-BP86/SDD/6-311G (d,p).

Bond/Angle	Length (Å)/Angle (°)		
	CC	CN	NN
Ir-C1	2.02	2.04	2.12
Ir-C2	2.02	2.03	2.11
Ir-N1	2.09	2.22	2.10
Ir-N2	2.08	2.07	2.08
Ir-S1	2.58	2.56	2.40
Ir-S2	2.60	2.43	2.44
S1-Ir-N1	92.4	90.1	165.5
S1-Ir-N2	92.5	83.3	84.4
S1-Ir-C1	83.8	165.5	89.2
S1-Ir-S2	100.8	100.5	99.5
S2-Ir-N1	84.2	83.9	86.8
S2-Ir-N2	98.8	175.8	175.2
S2-Ir-C2	86.3	98.4	98.1
C1-Ir-N1	97.4	78.0	78.3
C1-Ir-N2	79.2	90.2	98.0
C1-Ir-C2	89.5	94.4	171.9
C2-Ir-N1	79.3	172.0	94.3
C2-Ir-N2	95.3	79.2	78.5

Table S4. Selected bond lengths (Å) and angles (˚) for isomers of **1** in the lowest triplet state, as optimized with U-BP86/SDD/6-311G (d,p).

Bond/Angle	Length (Å)/Angle (˚)		
	CC	CN	NN
Ir-C1	2.01	2.03	2.08
Ir-C2	2.02	2.02	2.11
Ir-N1	2.10	2.04	2.14
Ir-N2	2.05	2.22	2.40
Ir-S1	2.61	2.45	2.42
Ir-S2	2.63	2.60	2.44
S1-Ir-N1	86.1	176.4	168.4
S1-Ir-N2	97.4	84.0	84.1
S1-Ir-C1	177.1	98.3	92.1
S1-Ir-S2	98.9	99.9	98.0
S2-Ir-N1	91.9	83.6	89.5
S2-Ir-N2	92.8	90.4	175.2
S2-Ir-C2	169.4	95.5	102.6
C1-Ir-N1	95.7	90.1	78.0
C1-Ir-N2	80.5	78.2	86.0
C1-Ir-C2	91.7	95.5	156.9
C2-Ir-N1	79.3	80.2	92.2
C2-Ir-N2	95.5	173.1	72.8

The stability of the triplets follows that of the lowest singlet states: the CC isomer is the most stable, NC 6.07 kJ mol^{-1} higher, and NN 37.92 kJ mol^{-1} higher.

Table S5. One-photon excitation data for **1** (CC isomer), obtained with TD-B3LYP/SDD/def2-TZVPP.

State	Energy/eV	Wavelength/nm	Osc. Strength	Character
T1	2.168	572.0	0	MLCT
T2	2.211	560.7	0	MLCT
S1	2.610	475.0	0.0279	MLCT
S2	2.698	459.6	0.0359	MLCT
S3	3.034	408.6	0.0113	Mixed
S4	3.154	393.1	0.0105	Mixed
S5	3.173	390.7	0.0160	MLCT

Table S6. One-photon excitation data for **2** (CC isomer)**,** obtained with TD-B3LYP/SDD/def2-TZVPP.

State	Energy/eV	Wavelength/nm	Osc. Strength	Character
T1	2.4714	501.7	0	MLCT
T2	2.4888	498.2	0	MLCT
S1	2.7103	457.5	0.0310	MLCT
S2	2.7571	449.7	0.0002	MLCT
S3	3.1595	392.4	0.0264	MLCT
S4	3.1811	389.8	0.0025	MLCT
S5	3.2599	380.3	0.0191	MLCT

Table S7. Photophysical data for **1** and **2**.

Complex	λ_{abs}[nm] (ε[$M^{-1}cm^{-1}\times10^4$])[a]	λ_{em}[nm][a]	Φ_{em}[b]		Lifetimes τ[ns][c]		$\Phi(^1O_2)$[d]	
			Air	N_2	Air	N_2	Indirect	Direct
**1**	445(1.5)	596	0.032	0.069	241	389	0.73	0.70
**2**	475(2.2); 550(1.1)	620	0.049	0.097	53	109	0.81	0.82

[a] Absorption and emission spectra recorded in PBS (containing 2% DMSO) at ambient temperature; [b] Φ_{em}, luminescence quantum yield in PBS (containing 2% DMSO); [c] τ, lifetime, determined in PBS (containing 2% DMSO). [d] $\Phi(^1O_2)$ determined by indirect and direct methods for 465 nm LED light irradiation. $Ru(bpy)_3]^{2+}$ was used as a standard photosensitizer ((Φ_{em} = 0.028 , $\Phi(^1O_2)$ = 0.22 in water[5]).

Table S8. (Photo)cytotoxicity (IC_{50}, μM) of the compounds towards 2D and 3D human A549 lung cancer and healthy MRC-5 lung cells.

Comp[b]	IC_{50} (μM)/ (PI)[a]									
	2D A549		2D MRC-5		3D A549 spheroids			3D MRC-5 spheroids		
	Dark	465nm	Dark	465nm	Dark	465nm	750nm	Dark	465nm	750nm
1	21.2±1.2	0.12±0.04 (177)	48.9±5.6	44.5±2.4 (1.1)	30.8±1.4	0.8±0.1 (39)	0.20±0.03 (204)	54.6±0.9	46.9±3.5 (1.2)	40.7±3.6 (1.3)
2	>100	0.3±0.1 (>333)	>100	>100	>100	1.0±0.2 (>100)	0.23±0.10 (>434)	>100	>100	>100
5-ALA	>100	>100	>100	>100	>100	>100	n.d.	>100	>100	n.d.
Cis-Pt	>50	>50	>50	>50	>50	>50	n.d.	>50	>50	n.d.

[a] 2D Cells or 3D spheroids incubated with the compounds for 2 h, medium replaced in both 'dark' and 'light' plates with fresh non-drug medium, 'light' plates irradiated with blue 96-array LEDs (465 nm, 2.88 J/cm^2) or 2-photon laser beam (750 nm, 10 J/cm^2, 100 fs). All plates incubated for another 46 h. The semidiameters of the spheroids were ~400 μm. 1P-PI and 2P-PI values in brackets are photocytoxicity indexes for 1-photon (465 nm) or 2-photon (750 nm) irradiation; PI = IC_{50} (dark) / IC_{50} (light); n.d. = Not determinable. [b] Compound; 5-ALA = 5-amino-levulinic acid; Cis-Pt = cisplatin.

Table S9. t-test results for the lysozyme peptide (FESNFNTQATNR, 714.8365 *m/z*).

**Sample**	**p value**
Drug treated A549 cancer cell line (dark)	0.16
Drug treated A549 cancer cell line (light)	0.26

Table S10. Identification and changes in cellular levels of oxidized peptides detected by MS in 5 triplicate samples, drug-treated non-irradiated versus drug-treated irradiated. Peptides labelled in green are up-regulated, in red down-regulated, and black indicates peptides with p values > 0.05.

Protein Entry	Protein	Peptide Sequence	P Value	Fold of Change	Modification
A0A0G2JIW1	HSP 70	AQIHDIVLVGGSTR	0.0029	5.80[a]	Oxidation (H)
E9PCX2	Aldose Reductase	YKPAVNQIECHPYLTQEK	0.0013	3.00[b]	Oxidation (H)
A6NL76	Actin, muscle	QEYDEAGPSIVHR	0.016	0.34	Oxidation (H)
Q9BQE3	Tubulin alpha chain	RAFVHWYVGEGMEEGEFSEAR	0.074	0.65	Oxidation (H, W, M)
P04350	Tubulin beta chain	GHYTEGAELVDAVLDVVR	0.46	1.21	Oxidation (H)

[a] Fold change = Area ratio (dark)/area ratio (light) = 0.29±0.01/0.05±0.01 for m/z = 741.4156

[b] Fold change = Area ratio (dark)/area ratio (light) = 0.45±0.15/0.15± 0.03 for m/z = 745.3786

Table S11. Mass errors for fragments detected from HSP70 peptide Ala329-Arg342 (containing Oxo-His332) by FTICR-MS. For definition of fragments, see Fig. 3b.

Assignment	Theoretical m/z	Observed m/z	Error/ ppm
$[y4]^{1+}$	420.220123	420.22018	0.14
$[b4+O]^{1+}$	466.240858	466.24092	0.13
$[y5]^{1+}$	477.241586	477.24147	-0.24
$[y6]^{1+}$	576.31	576.31012	0.21
$[y11+O-H_2O]^{2+}$	576.312012	576.31199	-0.04
$[b5+O]^{1+}$	581.267801	581.26783	0.05
$[y7]^{1+}$	689.394064	689.39433	0.39
$[b6+O]^{1+}$	694.351865	694.35161	-0.37
$[y8]^{1+}$	788.462478	788.46226	-0.28
$[b7+O]^{1+}$	793.420279	793.41971	-0.72
$[y9]^{1+}$	901.546542	901.54651	-0.04
$[b8+O]^{1+}$	906.504343	906.50455	0.23
$[b9+O]^{1+}$	1005.572757	1005.57262	-0.14
$[y10]^{1+}$	1016.573485	1016.57398	0.49
$[b10+O]^{1+}$	1062.594221	1062.59413	-0.09
	Absolute average		0.23
	Standard deviation		0.30

Table S12. Mass errors for fragments from aldose reductase peptide Tyr178-Lys195* (containing Oxo-His188) detected by FTICR-MS. For definition of fragments, see Fig. 3d.

Assignment	Theoretical m/z	Observed m/z	Error/ ppm
$[y3]^{1+}$	404.213975	404.21391	-0.16
$[y4]^{1+}$	505.261653	505.26167	0.03
$[y5]^{1+}$	618.345717	618.34585	0.22
$[M+3H]^{3+}$	745.368528	745.36849	-0.05
$[y6]^{1+}$	781.409046	781.4088	-0.31
$[y7]^{1+}$	878.46181	878.46207	0.30
$[y15+O]^{2+}$	923.443625	923.44316	-0.50
$[y16+O-H_2O]^{2+}$	962.964725	962.96462	-0.11
$[y16+O]^{2+}$	971.970007	971.97022	0.22
$[b11+O]^{1+}$	1356.636497	1356.63611	-0.29
		Absolute average	0.22
		Standard deviation	0.26

*Cys 187 in the peptide is alkylated by iodoacetamide.

Table S13. Quantification of proteins in the glycolytic pathway, the level of 22 peptides from 9 proteins increase significantly as a result of 465 nm light irradiation of A549 human lung cancer cells treated with complex **2**.

Peptide	p value	Protein[a]	Fold Change	Average Change[b]
IQQLEALQR	0.005007855	ALDH	3.35	3.35
LNVTEQEKIDK	0.007446963	AE	3.83	3.83
GILAADESTGSIAKR	0.000919911	FBA	5.25	5.25
HFVALSTNTTK	9.79498E-05	GPI	4.35	4.35
VVDLMAHMASKE	0.030391851	GAPDH	2.42	2.42
IVADKDYSVTANSK	0.00819089	L-LDH	1.97	2.11
VIGSGCNLDSAR	0.012997025	L-LDH	2.00	
VTLTSEEEAR	0.000428796	L-LDH	2.81	
DLADELALVDVIEDKLK	0.018006519	L-LDH	2.36	
AHSSMVGVNLPQK	0.003594354	PGK	2.98	2.98
AGKPVICATQMLESMIK	0.005024276	PK	3.79	3.47
FGVEQDVDMVFASFIR	0.020145353	PK	3.56	
GSGTAEVELKK	0.038292593	PK	2.04	
GVNLPGAAVDLPAVSEKDIQDLK	0.009882131	PK	10.59	
ITLDNAYMEK	0.009179796	PK	3.96	
LNFSHGTHEYHAETIK	0.000836253	PK	3.60	
RFDEILEASDGIMVAR	0.030084641	PK	2.02	
TATESFASDPILYRPVAVALDTK	0.033109078	PK	2.11	
IAVAAQNCYK	0.001460703	TPI	6.28	4.40
IIYGGSVTGATCK	0.011379276	TPI	1.79	
SNVSDAVAQSTR	0.000220343	TPI	4.82	
VVLAYEPVWAIGTGK	0.002355749	TPI	6.49	

[a] ALDH: Aldehyde dehydrogenase; AE: Alpha-enolase; FBA: Fructose-bisphosphate aldolase; GPI: Glucose-6-phosphate isomerase; GAPDH: Glyceraldehyde-3-phosphate dehydrogenase; L-LDH: L-lactate dehydrogenase; PGK: Phosphoglycerate kinase; PK: Pyruvate kinase; TPI: Triosephosphate isomerase. [b] Weighted average.

Figures

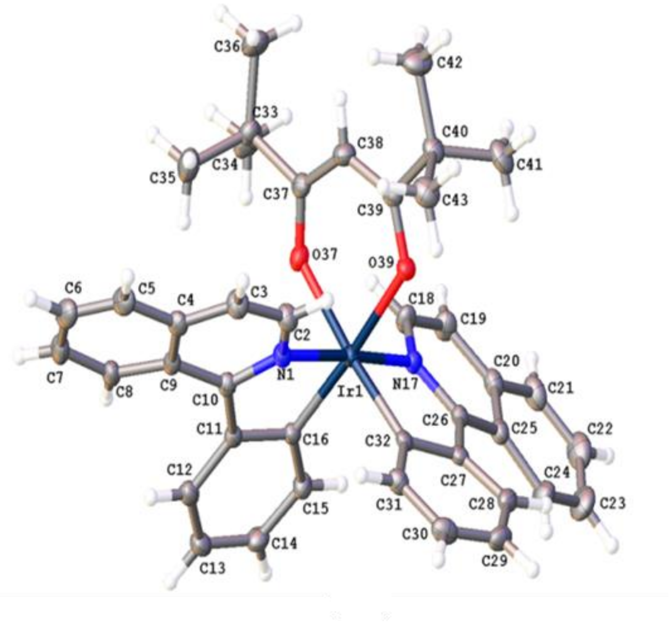

X-ray crystal structure of complex 2

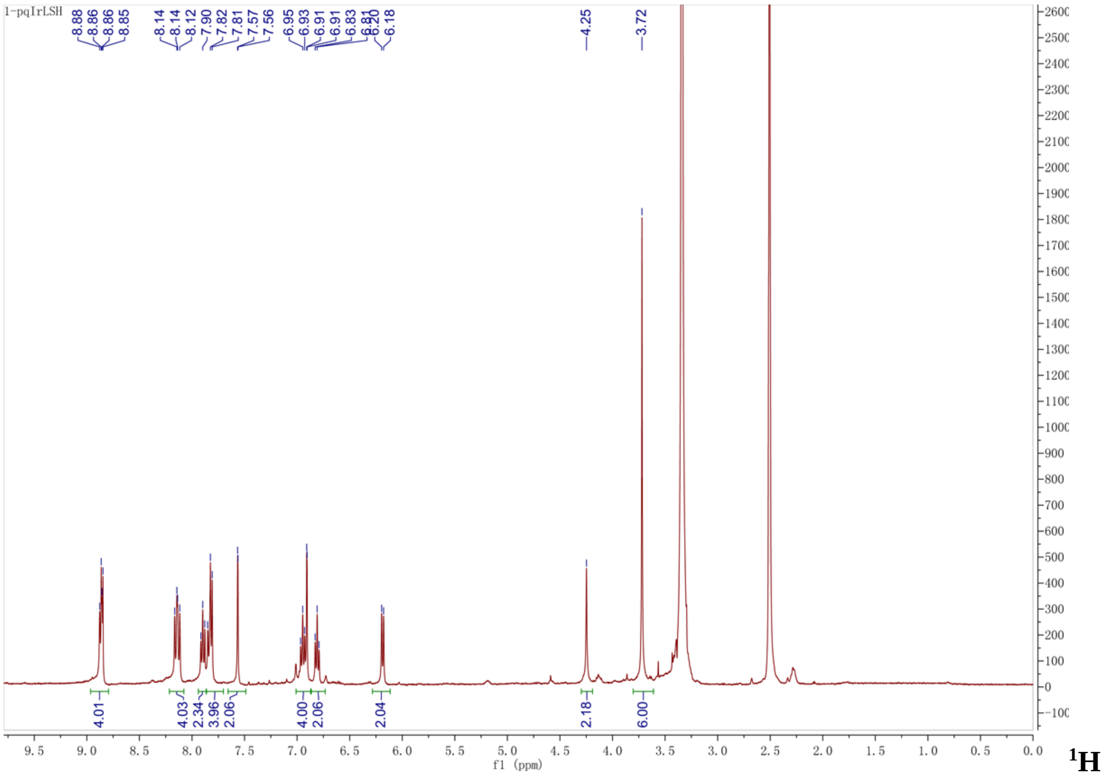

NMR of Complex 1 in d_6-DMSO

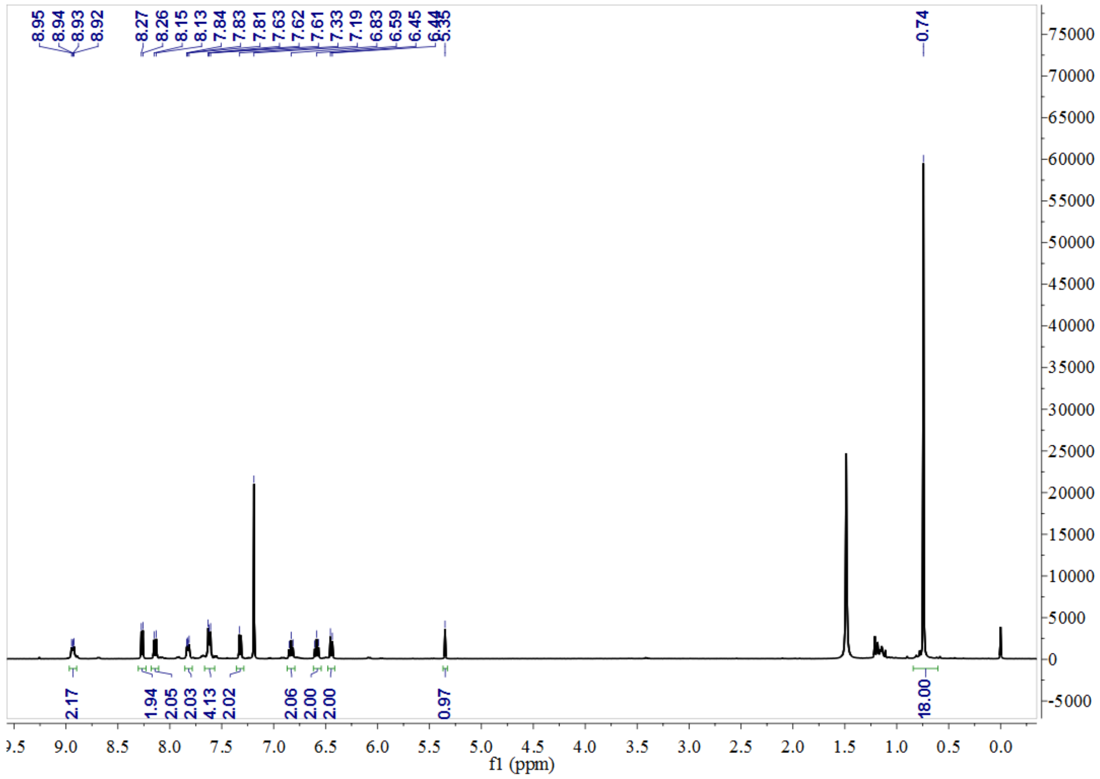

^{1}H NMR of Complex 2 in $CDCl_3$

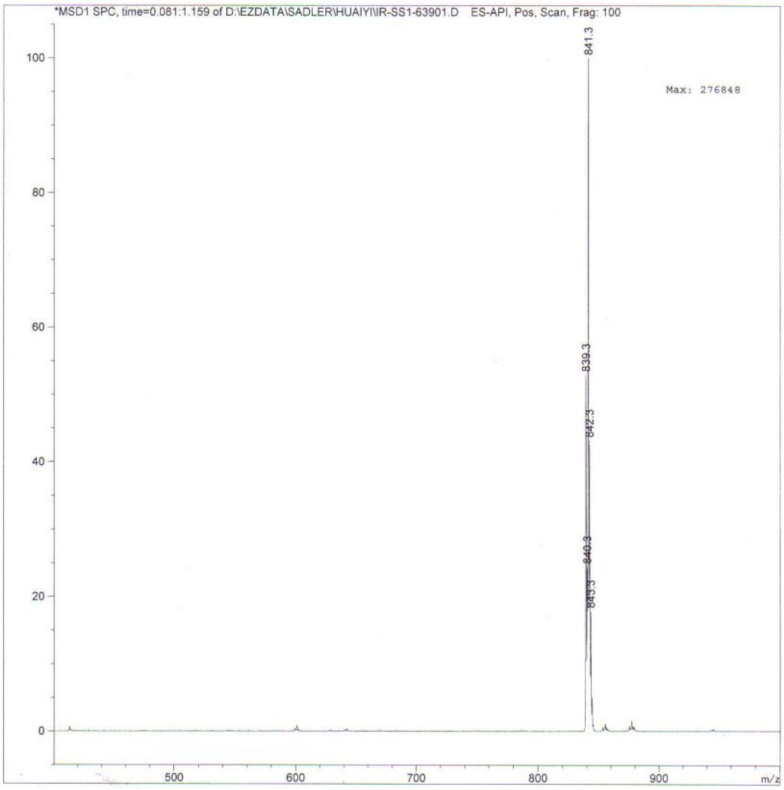

ESI-MS of Complex 1: 841.3 M[**1**-Cl]$^{+}$.

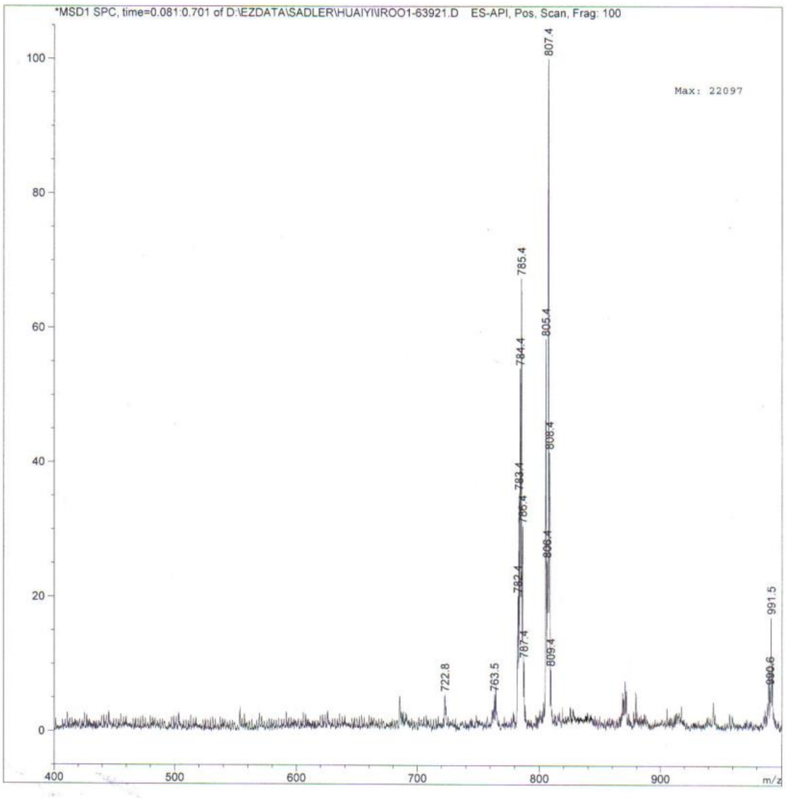

ESI-MS of Complex 2: 785.4 (M[**2**]), 808.4 M ([**2**+Na]$^{+}$.

Figure S1. The X-ray crystal structure of complex **2**, together with 400 MHz ^{1}H NMR and mass spectra of complexes **1** and **2**.

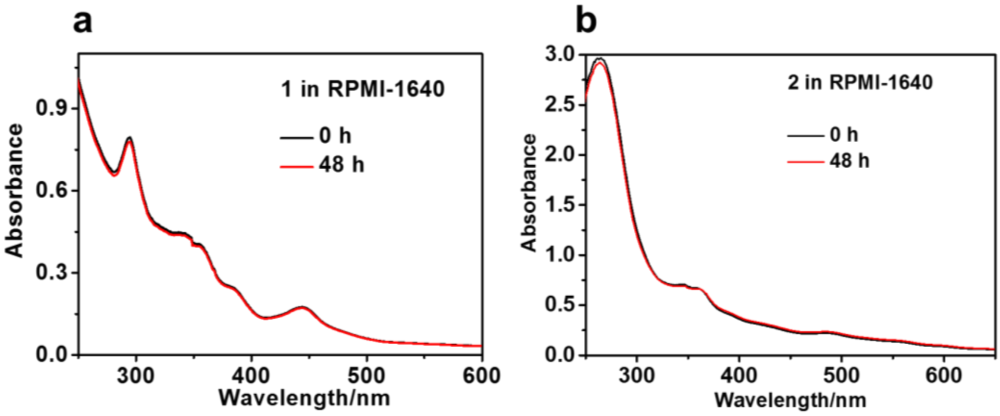

Figure S2. UV-vis absorption spectra for (a) complex **1** and (b) complex **2** in RPMI-1640 cell culture medium after 0 h and 48 h at 310 K, showing that the complexes are stable.

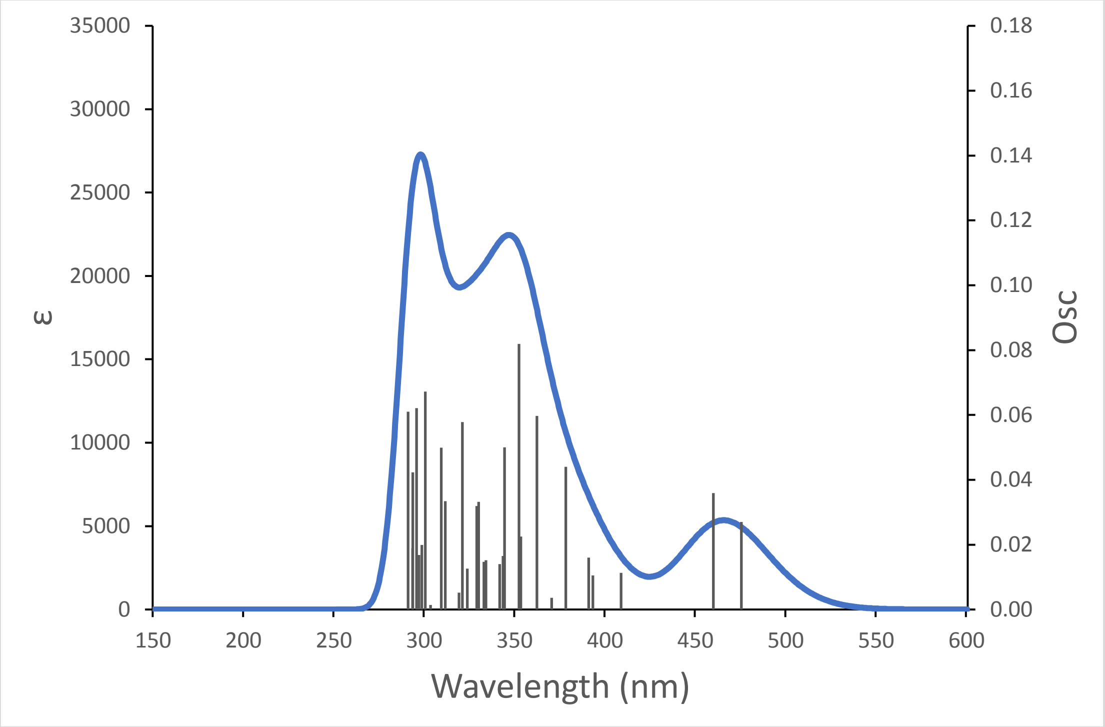

Figure S3. Simulated electronic spectrum for **1** (CC isomer) obtained with TD-B3LYP/SDD/6-311G (d,p).

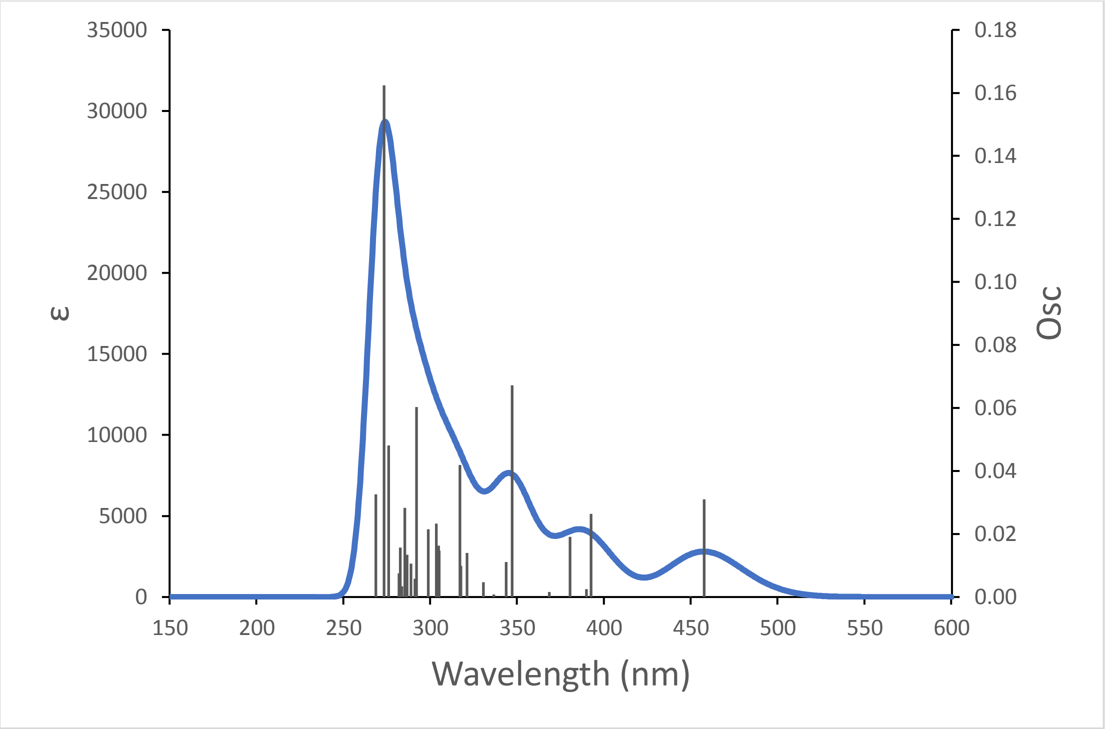

Figure S4. Simulated electronic spectrum for **2** (CC isomer) obtained with TD-B3LYP/SDD/6-311G (d,p).

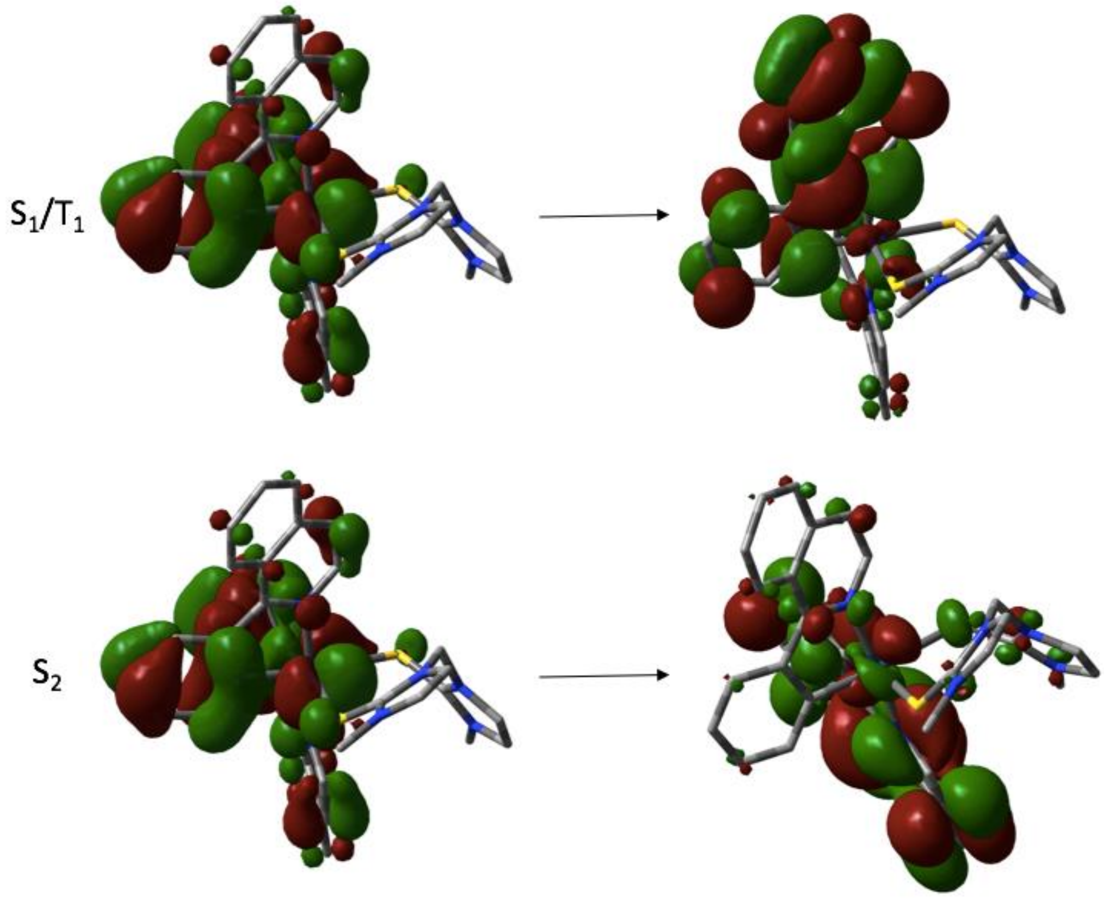

Figure S5. Dominant canonical particle-hole orbitals characterising the two lowest singlets and first triplet state of **1** (CC isomer), obtained with TD-B3LYP/SDD/6-311G (d,p).

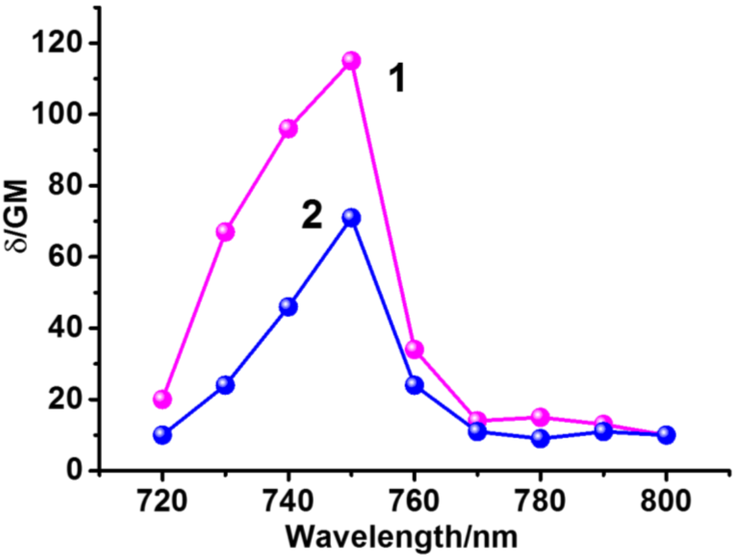

Figure S6. Two-photon absorption cross-sections of **1** and **2** at excitation wavelengths between 720 and 800 nm.

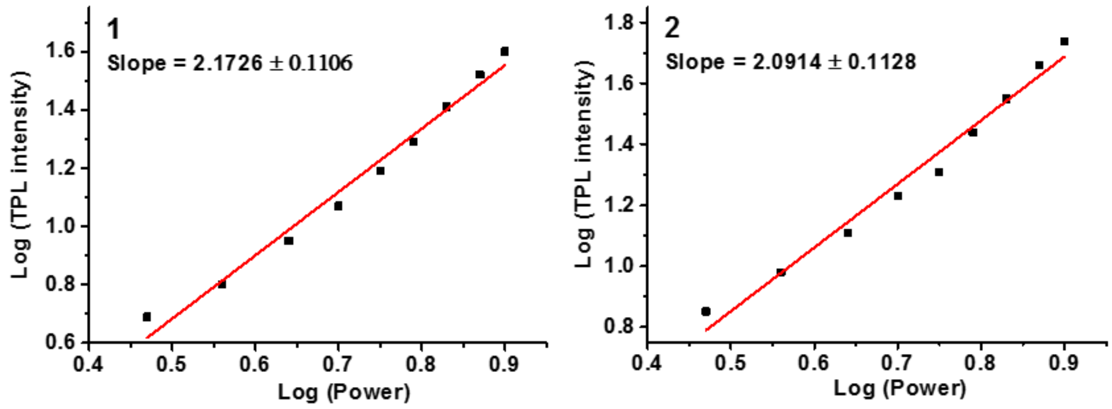

Figure S7. Logarithmic two-photon luminescence intensity of the iridium(III) complexes as a function of the logarithmic power using the wavelength of the laser with largest TPA. The two-photon excitation active process was confirmed by its power dependence: a linear log-log relationship between the emission intensity and incident power with a gradient n ≈ 2.

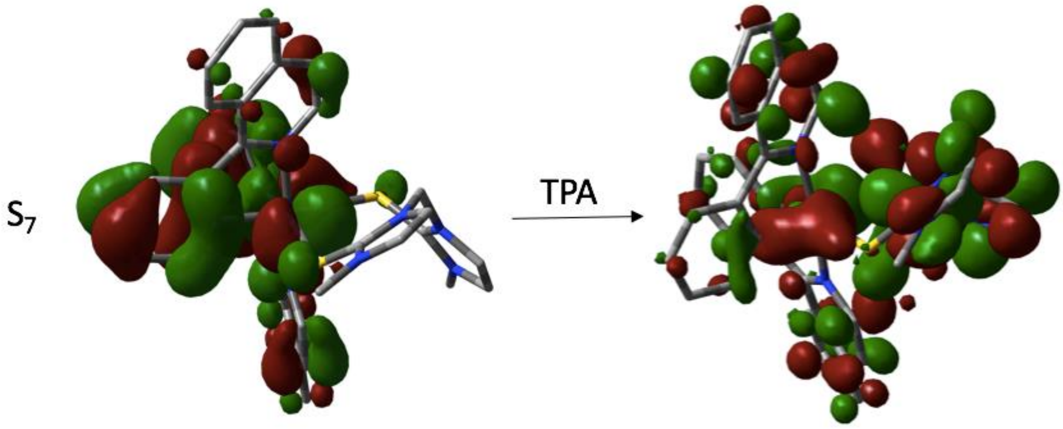

Figure S8. Dominant canonical particle-hole orbitals characterising the very strong two-photon absorption to S_7 in **1** (CC isomer), obtained with ATDA-TD-B3LYP/SDD/6-311G (d,p).

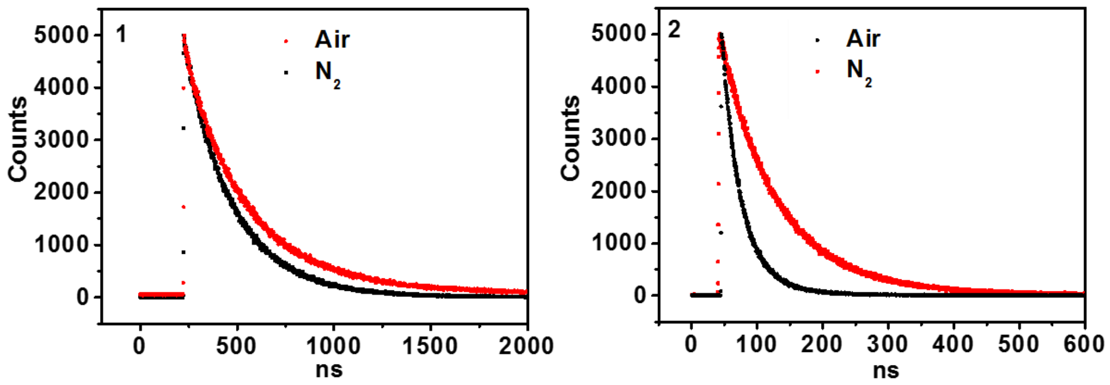

Figure S9. Phosphorescence lifetimes of iridium(III) complexes **1** (left) and **2** (right) measured in air and N_2.

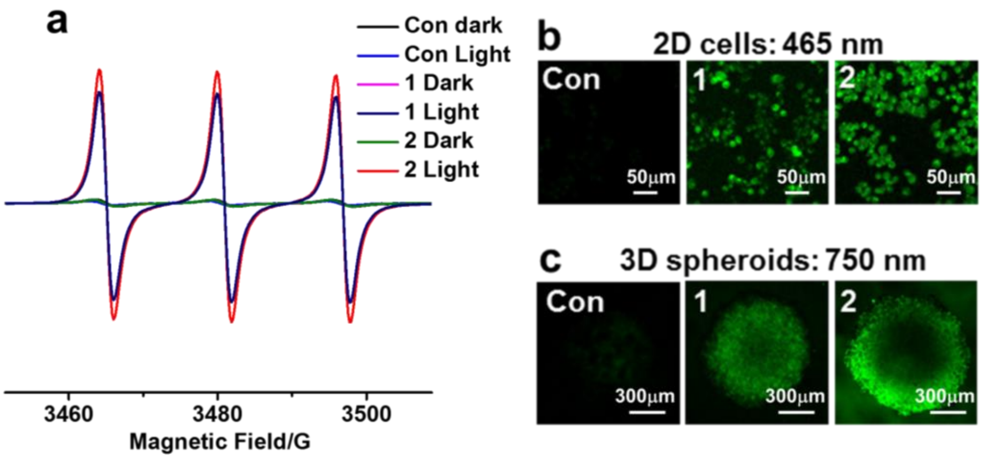

Figure S10. Singlet oxygen generation. (a) EPR signals from **1**, **2** and control in the dark and under 465 nm irradiation for 10 min. (b) 2D A549 monolayer cells treated with the Ir(III) complexes and DCFH-DA in the dark, washed with PBS and then irradiated with 465 nm light (10 min). (c) 3D A549 spheroid tumors were incubated with the Ir complexes (10 μM) for 2 h, stained with DCFH-DA (10 μM, 1 h) in the dark, transferred to fresh PBS, and irradiated with 750 nm two-photon laser. Fluorescence images were recorded by confocal laser scanning microscopy (λ_{ex} = 488 nm, λ_{em} = 530 ± 20 nm).

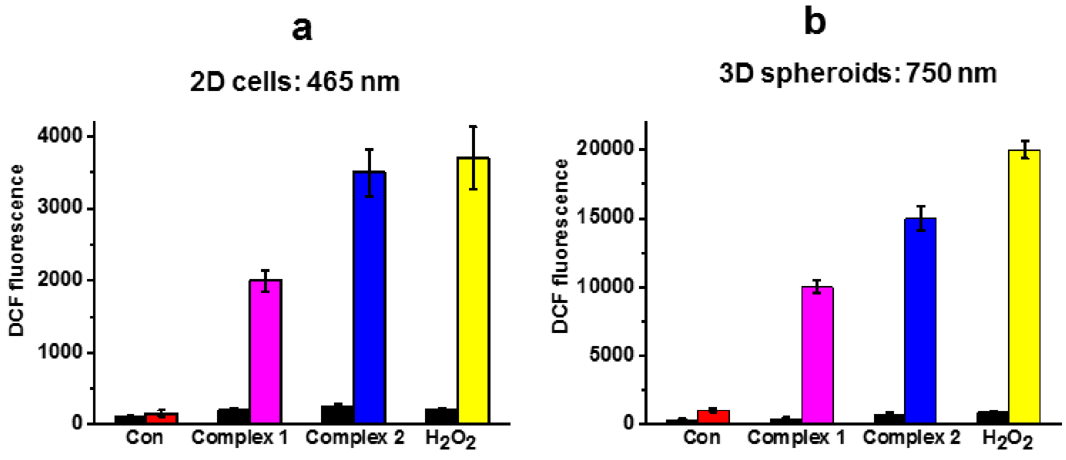

Figure S11. ROS measurements in cells. (A) A549 monolayer cells and (B) 3D A549 spheroid tumors were incubated with the iridium complexes (10 μM) for 2 h, and then stained with DCFH-DA (10 μM, 1 h) in the dark. Then the monolayer cells were irradiated with 465 nm light and the 3D spheroid tumors were irradiated upon 750 nm two-photon. The DCF florescence intensities were detected by Promega microplate reader (λ_{ex} = 488 nm, λ_{em} = 530±20 nm). Dark bars for the cells in the 'dark' and colored bars for the cells under irradiation.

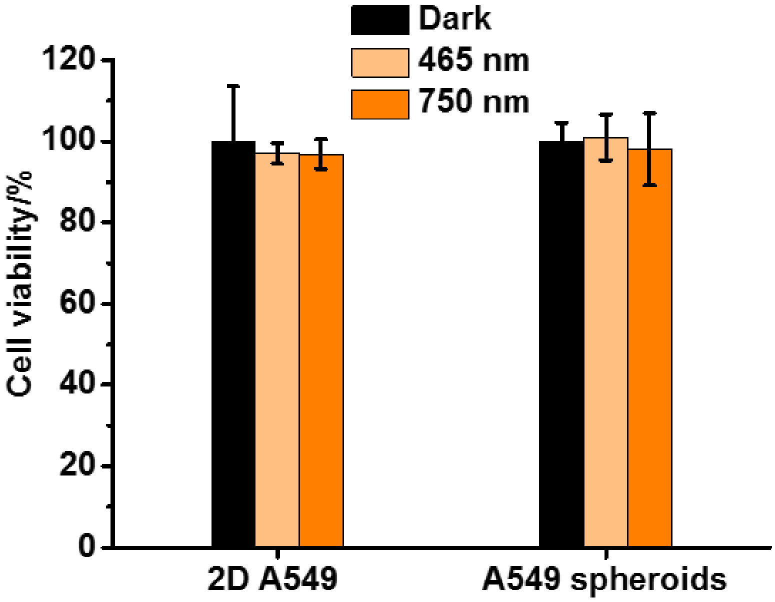

Figure S12. Effect of irradiation with blue 96-array LEDs for 10 min (465 nm, 2.28 J/cm^2) and two-photon laser irradiation (750 nm, 10 J/cm^2) on the viability of control A549 cells.

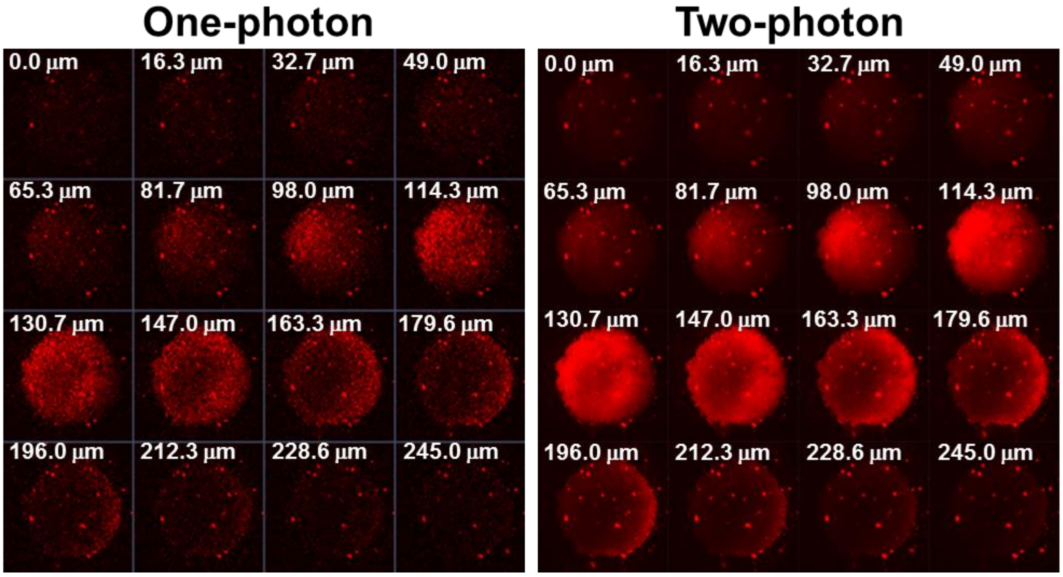

Figure S13. 1- and 2-photon Z-stack images of 3D tumor spheroids treated with complex **2** (as shown in Fig. 2) captured every 16.3 μm along the Z-axis.

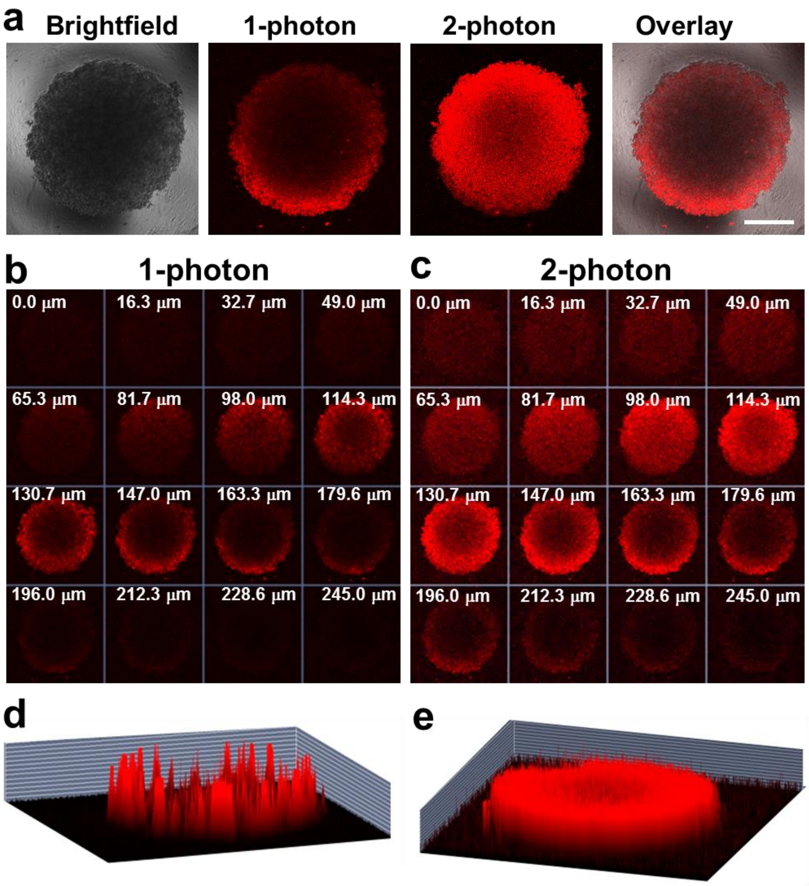

Figure S14. Phosphorescence imaging of A549 spheroids for complex **1**. The spheroids were incubated with complex **1** (10 μM) for 2 h. (a) Comparison of brightfield, one-photon (λ_{ex} = 458 nm), and two-photon (λ_{ex} = 750 nm) excitation, λ_{em} = 600 ± 30 nm. (b) 1-photon and 2-photon (c) Z-stack images were taken every 16.3 μm from the top to bottom of the spheroids. (d, e) 3D Z-stack images from (b) and (c), respectively. Images were taken under a 10× objective.

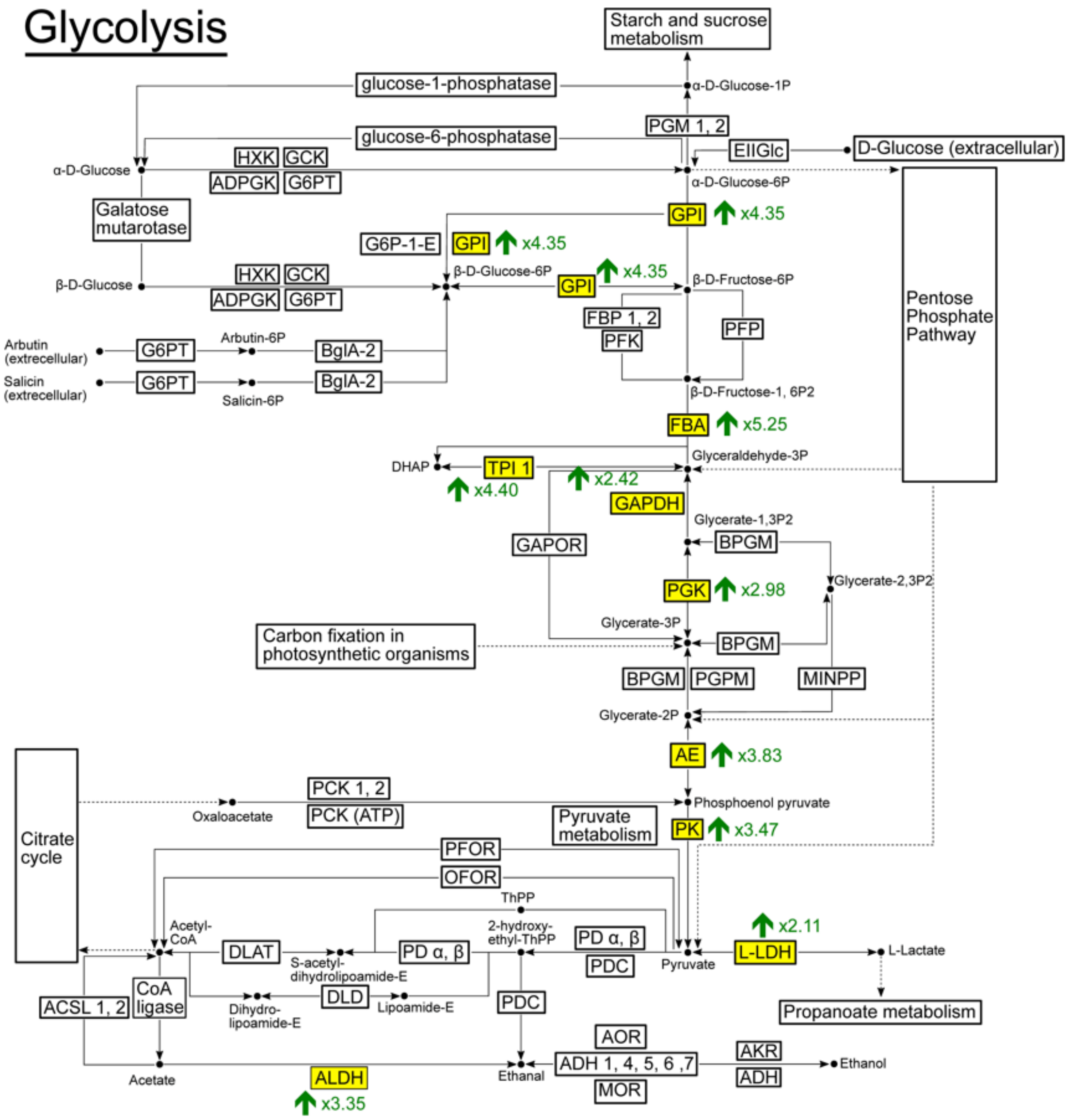

Figure S15. Effects of irradiation of complex 2 on the levels proteins associated with glycolysis pathway in A549 lung cancer cells. 9 upregulated proteins highlighted in yellow were identified by LC-MS/MS in our experiment, with the magnitude of the fold-change indicated in green.

Key: **PGM**: Phosphoglucomutase; **EIIGlc**: D-glucose Npi-phosphotransferase; **HXK**: Hexokinase; **GCK**: Glucokinase; **ADPGK**: ADP dependent glucokinase; **G6PT**: D-glucose-6-phosphotransferase; **G6P-1-E**: D-glucose-6-phosphate 1-epimerase; **GPI**: Glucose-6-phosphate isomerase; **BgIA-2**: 6-phospho-beta-D-glucosyl-D-glucose glycohydrolase; **FBP**: Fructose-bisphosphatase; **PFK**: Phosphofructokinase; **PFP**: D-fructose-6-Phosphate 1-phosphotransferase; **FBA**: Fructose-bisphosphate aldolase; **TPI**: Triosephosphate isomerase; **GAPDH**: Glyceraldehyde-3-phosphate dehydrogenase; **GAPOR**: D-glyceraldehyde-3-phosphate: $NADP^+$ oxidoreductase; **BPGM**: Bisphosphoglycerate mutase; **PGK**:

Phosphoglycerate kinase; **PGPM**: D-phosphoglycerate 2,3-phosphomutase; **MINPP**: Multiple inositol-polyphosphate phosphatase; **AE**: Alpha-enolase; **PCK**: Phosphoenolpyruvate carboxykinase; **PCK (ATP)**: ATP Oxaloacetate carboxy-lyase; **PK**: pyruvate kinase; **PFOR**: Pyruvate ferredoxin-2-oxidoreductase; **OFOR**: 2-oxocarboxylate: ferredoxin-2-oxidoreductase; **ACSL**: Acyl-CoA-synthetase; **DLAT**: Dihydrolipoamide S-acetyltransferase; **DLD**: Dihydrolipoamide dehydrogenase; **PD**: Pyruvate dehydrogenase; **PDC**: 2-oxo-acid-carboxyl-lyase; **L-LDH**: Lactate dehydrogenase; **ALDH**: Aldehyde dehydrogenase; **ADH**: Alcohol dehydrogenase; **AOR**: Alcohol cytochrome c oxidoreductase; **MOR**: Methanol cytochrome c oxidoreductase; **AKR**: Aldo-keto reductase.

References

[1] Y. Chen, et al. *Biomaterials* **2014,** *35,* 2.

[2] O. V. Dolomanov, et al. *J. Appl. Cryst.* **2009,** *42,* 339.

[3] G. M. Sheldrick, *Acta Cryst.* **2015,** *A71,* 3.

[4] P. Cronstrand, et al. *Chem. Phys. Lett.* **2002,** *352,* 262.

[5] I. H. Nayyar, et al. *J. Phys. Chem. C* **2013,** *117*, 18170.

[6] I. A. Mikhailov, S. Tafur, A. E. Masunov, *Phys Rev A: At Mol. Opt. Phys.* **2008,** *77,* 012510.

[7] M. J. T. Frisch, et al. *Gaussian 09,* **2009,** Revision D.01.

[8] C. Mari, et al. *J. Chem. Eur.* **2014,** *20,* 14421.

[9] D. I. Pattison, A. S. Rahmanto, M. J. Davies, *Photochem. Photobiol. Sci.* **2012,** 11, 38.

[10] M. J. Davies, *Biochem Biophys. Res. Commun*. **2003,** *305,* 761.

[11] D. W. Huang, B. T. Sherman, R. A. Lempicki, *Nat. Protoc.* **2009,** *4,* 44.

[12] M. Kanehisa, M. Furumichi, M. Tanabe, Y. Sato, K. Morishima, *Nucleic Acids Res.* **2017,** *45,* D353–D36

[13] M. Kanehisa, Y. Sato, M. Kawashima, M. Furumichi, M. Tanabe, *Nucleic Acids Res.* **2016,** *44,* D457.

[14] M. Kanehisa, S. Goto, *Nucleic Acids Res.* **2000,** *28,* 27.
